# Dynamin-2 R465W mutation induces long range perturbation in highly ordered oligomeric structures

**DOI:** 10.1038/s41598-020-75216-0

**Published:** 2020-10-23

**Authors:** Fernando Hinostroza, Alan Neely, Ingrid Araya-Duran, Vanessa Marabolí, Jonathan Canan, Maximiliano Rojas, Daniel Aguayo, Ramón Latorre, Fernando D. González-Nilo, Ana M. Cárdenas

**Affiliations:** 1 Centro Interdisciplinario de Neurociencia de Valparaíso, Facultad de Ciencias, Universidad de Valparaíso, Gran Bretaña 1111, Valparaiso, Chile; 2 Centro de Investigación de Estudios Avanzados del Maule (CIEAM), Vicerrectoría de Investigación y Postgrado, Universidad Católica del Maule, Av. San Miguel 3605, Talca, Chile; 3 Center for Bioinformatics and Integrative Biology, Facultad de Ciencias de la Vida, Universidad Nacional Andrés Bello, Av. República 330, Santiago, Chile

**Keywords:** Computational biology and bioinformatics, Structural biology, Diseases, Neurology

## Abstract

High order oligomers are crucial for normal cell physiology, and protein function perturbed by missense mutations underlies several autosomal dominant diseases. Dynamin-2 is one of such protein forming helical oligomers that catalyze membrane fission. Mutations in this protein, where R465W is the most frequent, cause dominant centronuclear myopathy, but the molecular mechanisms underpinning the functional modifications remain to be investigated. To unveil the structural impact of this mutation in dynamin-2, we used full-atom molecular dynamics simulations and coarse-grained models and built dimers and helices of wild-type (WT) monomers, mutant monomers, or both WT and mutant monomers combined. Our results show that the mutation R465W causes changes in the interactions with neighbor amino acids that propagate through the oligomer. These new interactions perturb the contact between monomers and favor an extended conformation of the bundle signaling element (BSE), a dynamin region that transmits the conformational changes from the GTPase domain to the rest of the protein. This extended configuration of the BSE that is only relevant in the helices illustrates how a small change in the microenvironment surrounding a single residue can propagate through the oligomer structures of dynamin explaining how dominance emerges in large protein complexes.

## Introduction

Many physiological processes within the cell rely on large protein complexes for which correct protein–protein interactions and assembly are crucial for their proper function. Perturbations on these interactions alter protein functions leading to severe diseases. Helices formed by dynamins are highly ordered oligomeric structures essential for life, and mutations in these proteins disrupt endocytosis, one of the essential processes for cell survival, causing autosomal dominant diseases^1^. However, how missense mutations perturb helix conformations is poorly known.

**Figure 1:**
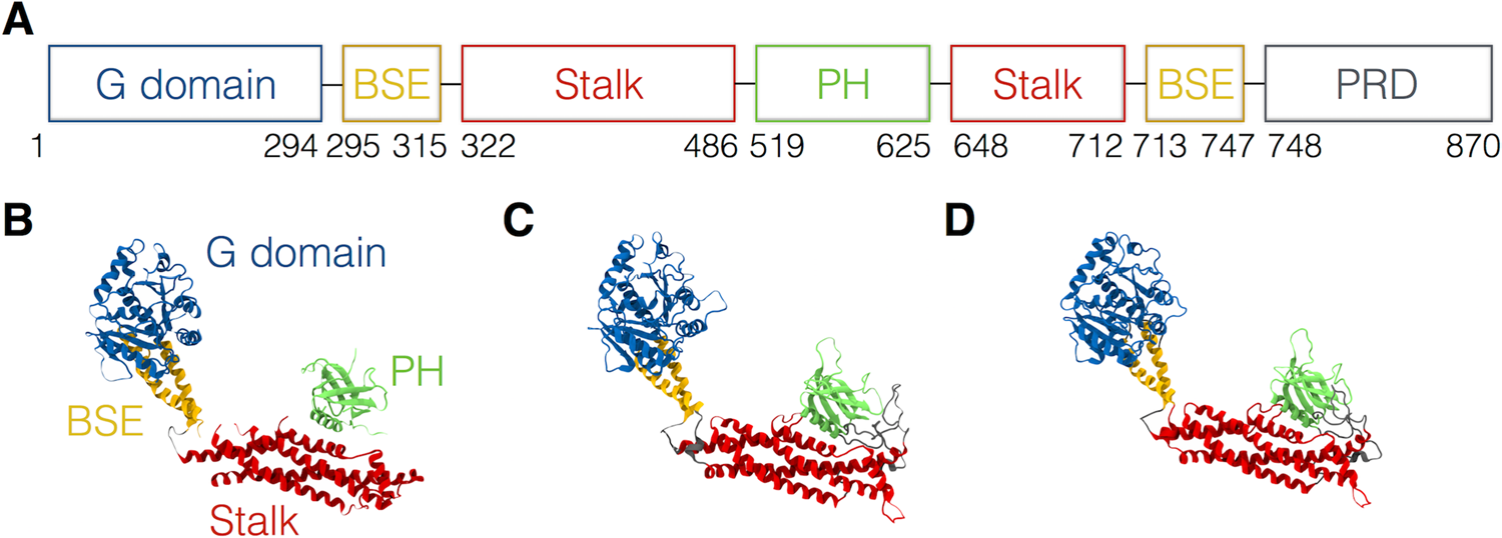
Dynamin domains. (**A**) Dynamin-2 domains. Numbers indicate the amino acid number in the protein. (**B**) Human Dyn-1 crystallographic structure solved by Faelber et al.^24^ (PDB ID: 3SNH) missing the proline-rich domain and loops. (**C**) Human dynamin-1 ⊗ PRD full-length model. (**D**) Homology model of the human dynamin-2. GTPase domain is in blue, bundle signaling element (BSE) is in yellow, stalk is in red, and pleckstrin homology (PH) domain is in green.

Dynamins are a family of large GTPase proteins represented by three members (dynamin 1, 2, and 3) that form higher-order oligomers and are involved in membrane remodeling^2^. They all share a common structure consisting of five domains: an N-terminal G domain that hydrolyzes GTP when it interacts with other G domains, a middle and a GTPase effector domains that compose the “stalk region” involved in oligomerization, a bundle signaling element (BSE) that connects the G-domain with the stalk, a pleckstrin homology domain (PH) that binds inositol phospholipids, and a C-terminal proline and arginine-rich domain (PRD) that mediates the interaction with SH3-domain-containing proteins^3^ (Fig. 1A,B). In the cytosol, dynamins are predominantly in a tetrameric conformation, exhibiting a basal GTPase activity^4^. In the presence of anionic lipid membrane surfaces, dynamins assemble into helices surrounding membrane cylinders^4,5^. In this configuration, the PH domains are inserted into the membrane, the stalk regions interact with the neighboring monomer in a crisscrossed fashion and the G‐domains are oriented outward^6^. It has been proposed that this dynamin conformation favors interaction between G domains of adjacent rungs and therefore GTP hydrolysis^7,8^. This GTP hydrolysis promotes the motion of the BSE that would transmit the conformational change to the rest of the molecule, producing the helix constriction^8–10^ that catalyzes membrane fission^4^. This mechanism is crucial for endocytosis^11^, vesicle budding from Golgi membranes^12^, T-tubule formation^13^ and mitochondrial division^14^. The three dynamin isoforms are implicated in endocytosis, but only dynamin-2 (dyn-2) participates in the other three aforementioned processes^12–14^. However, the mechanisms underlying these processes exclusive to dyn-2 are unclear and may involve cytoskeletal rearrangement^15–17^.

The correct function of higher-order oligomers depends on the proper interaction and binding between the proteins involved. Thus, to catalyze membrane fission during endocytosis, dynamin monomers must interact with other monomers in a precise manner. Therefore, perturbations in these interactions are likely to disrupt endocytosis. Indeed, several dyn-2 mutations causing centronuclear myopathy (CNM)^1,18,19^, disrupt endocytosis^20–22^. However, some of them, including the mutation R465W, can also retain and even increase fission activity in vitro, and promote membrane fragmentation^13^. This latter property seems to correlate with an increased stability of higher order oligomers^23^. Interestingly, the mutation R465W falls outside regions involved in oligomerization^20^, raising the question about how these peripheral structural changes can perturbs dynamin–dynamin interactions and/or helix organization.

New insights into the structural and dynamic properties of dynamin-dynamin interactions can be gained through molecular dynamics simulations (MD). Molecular simulation of the dynamin helix though constitutes nowadays an insurmountable challenge since it comprises several millions of atoms and a conformational change taking place in the scale of milliseconds. Coarse-grained (CG) molecular dynamics simulations solve this problem by reducing the numbers of particles in the systems, simplifying the analysis to low-frequency movements, like domain-domain interactions, and geometrical parameters related to the self-assembly events. Here, we took advantage of this method to determine the structural impact of the disease-causing R465W mutation in dyn-2 helices. We found that the exchange of the arginine by a tryptophan at this position impairs the interaction zones between dyn-2 monomers and favors an extended BSE conformation that impacts the helix structure and dynamics.

## Results and discussion

### R465W mutation reduces the free binding energy between dynamin-2 monomers

The dynamin dimer constitutes the structural unit of dynamin high-order oligomers^8,9,24,25^. Dynamin dimers identified in dynamin-1 (dyn-1) crystal structures are arranged in crisscrossed fashion connected via an interface (interface-2) located in the center of two stalk domains^9,24^. As human dyn-1 shares 81% of sequence identity with dyn-2, we built a dyn-2 homology model using the human dyn-1 crystallographic structure (PDB ID: 3SNH)^24^ as a template. The non-resolved regions were built using Modeller v9.10 software^26^ (Fig. 1B–D). Then, by using full-atom MD, we built three systems: a WT dimer, a heterodimer (HT), that contains a WT monomer and an R465W mutant monomer, and a full-mutant (FM) dimer composed of two mutant monomers (Suppl. Figure 1A–C). It should be noted here that in all dimers the hinge joining the BSE and stalk cover a larger range of conformations since there is no spatial restriction by other proteins.

**Figure 2:**
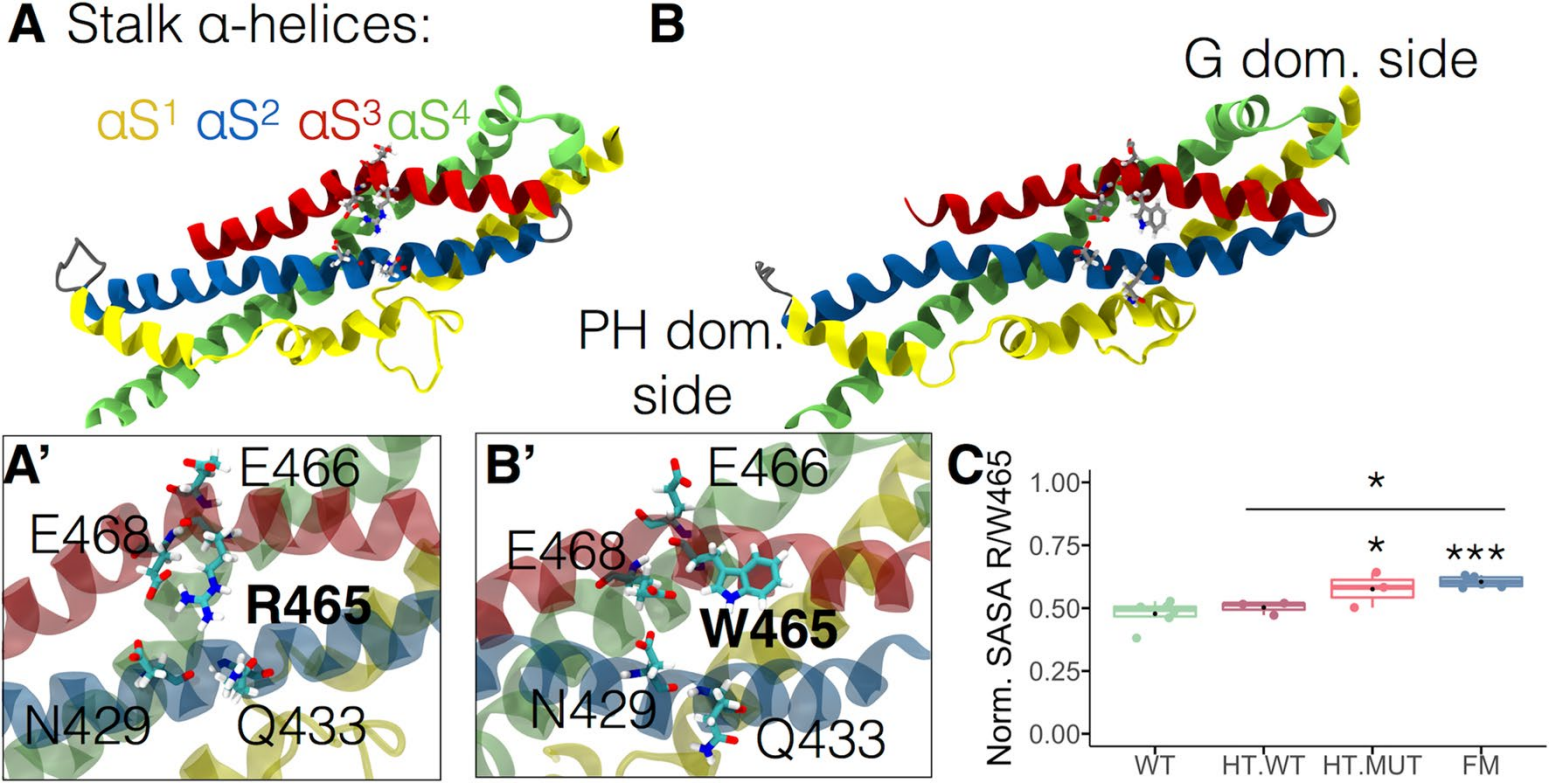
Impact of the R465W mutation on its local environment. (**A**) Stalk region of a WT dynamin monomer showing the interaction of R465 with N429, Q433, E466, and E468. (**A′**) Zoom of R465 in **A**. (**B**) Stalk region of a mutant monomer highlighting the lack of interaction of W465 with other amino acids. (**B′**) Zoom of W465 in **B**. (**C**) Each dot represents the average over the last 10 ns of the simulation of the change in normalized SASA. Boxes indicate 25–75 percentiles, lines within boxes indicate medians, and whiskers indicate minimum and maximum values. *p < 0.05; ***p < 0.001 compared with the WT dimer (one-way ANOVA and Turkey’s posttest).

Residue 465 is located halfway in helix αS3 within the four helical bundles forming the stalk (Fig. 2A). As shown in Fig. 2A,A′, R465 interacted through either hydrogen bonds or salt bridges with N429, Q433, E466, and E468 in the WT monomer of the WT dimer, as well as in the WT monomer of the HT dimer (Fig. 2A). These interactions are lost when R465 was replaced by tryptophan in both HT and FM dimers (Fig. 2B). Moreover, the Solvent Accessible Surface Area (SASA) was larger for residue W465 than R465 even in the mutant monomer of the HT dimer (Fig. 2C). Each SASA was normalized with the reported empirical SASA of each amino acid^27^.

In cells carrying the R465W mutation, dyn-2 forms abnormal cytoplasmic aggregates^23^. This alteration might be explained by the exposed W465 (Fig. 2B,B′), as solvent exposed tryptophans favor protein aggregation^28,29^. In fact, in arylamine *N*-acetyltransferases, the replacement of an arginine located in the peripheral region in the structure, by a tryptophan favors protein aggregation^28^. The authors propose that tryptophan might cause steric clashes with the surrounding residues, and its exposition to the solvent could change surface properties of the protein^27^. Tryptophan residues also contribute to the aggregation of the myoglobin, as consequence of the intrinsic propensity of this amino acid to establish hydrophobic interactions^29^.

**Figure 3:**
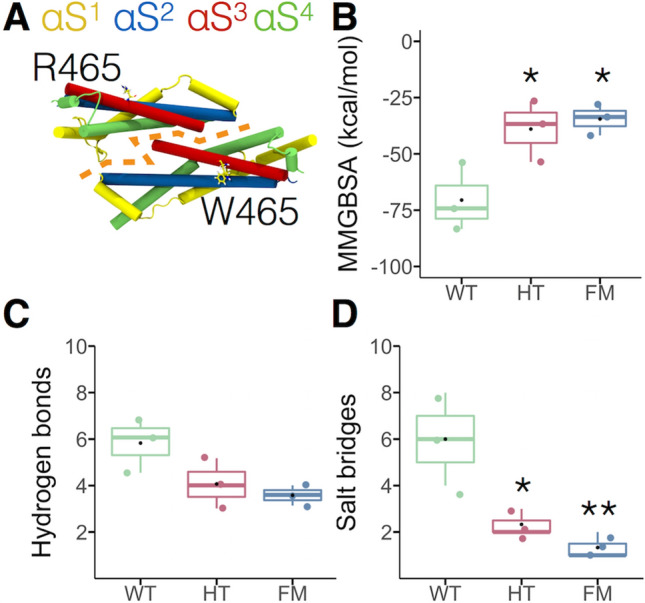
Impact of the R465W mutation on the free binding energy of dynamin-2 dimers. (**A**) Stalks in heteromeric dimer configuration. Each stalk is composed of four α-helices named from αS^1^ to αS^4^. Interaction between monomers involves alpha-helices αs2, αs3, and αs4. Orange dashed line represents interface-2. (**B**) Free binding energy of the dynamin-dynamin interaction calculated by molecular mechanics generalized Born surface area continuum solvation (MM/GBSA). (**C**) Hydrogen bonds number and (**D**) salt bridges number presents in the dynamin-dynamin interface-2. Dots represent the average values of the last 10 ns of simulation. Boxes indicate 25–75 percentiles, lines within boxes indicate medians, and whiskers indicate minimum and maximum values. *p < 0.05; **p < 0.01 compared with the WT dimer (one-way ANOVA and Turkey’s posttest).

We should bear in mind that these are dimers and thus we next focused on whether this mutation impacts the interaction between monomers involving alpha-helices αs2 to αs4 (Fig. 3A), described as interface-2 by Faelber et al.^24^. We quantified the free binding energy, hydrogen bonds and salt bridges of this interface. According to the Molecular Mechanics-Generalized-Born and Surface Area continuum solvation (MM/GBSA) calculations carried out during the last 10 ns of simulation, the WT dimer had a lower free binding energy in comparison to both HT and FM dimers (Fig. 3B). In agreement with the MM/GBSA results, both HT and FM dimers exhibited fewer hydrogen bonds in the interface-2 (Fig. 3C) and a significant reduction in the number of salt bridges (Fig. 3D). Residue 465 is not exposed to the interaction surface of neighboring monomers in all systems, WT, HT (see Fig. 3A) or FM dimers. However, the replacement of arginine by tryptophan at position 465 by perturbing the interaction with other residues indirectly destabilized the interaction between monomers through interface-2 as a clear example of allosteric propagation of the mutant-induced structural perturbations. This raises the question of whether this mutation may also have an impact on the helix structure.

### R465W mutation changes the helix tetramers’ configuration

**Figure 4:**
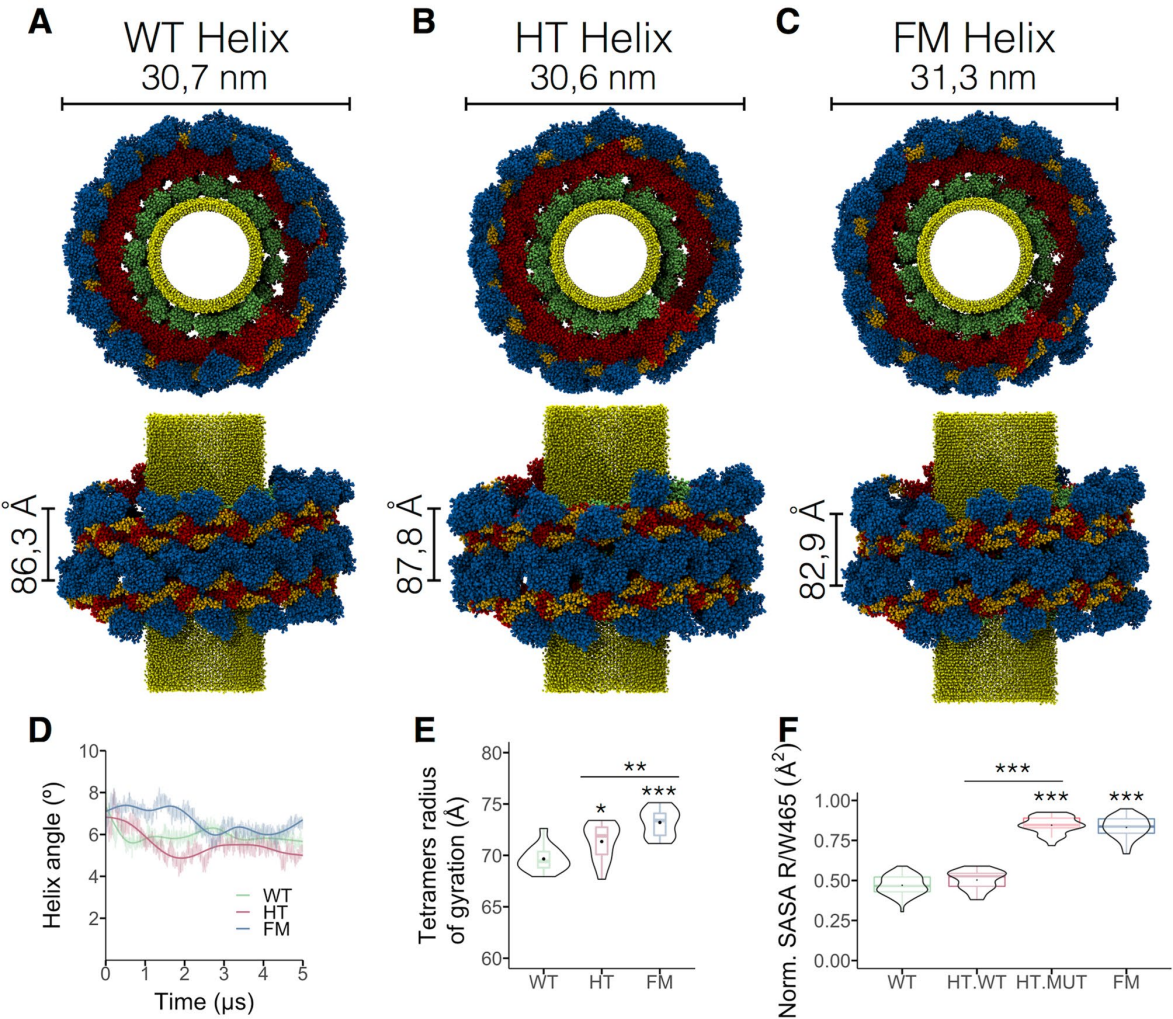
R465W mutation changes the helix tetramers’ configuration. Top view (x- and y-axes) and lateral view of the (**A**) WT, (**B**) HT, and (**C**) FM helices at the end of 5 μs of simulation. Helix diameter and pitch are indicated. G domains are in blue, BSEs in yellow, stalks in red, PH domains in green, lipid nanotube in light yellow. (**D**) Evolution of the helix angles during the 5 μs of simulation. (**E**) Radius of gyration of dyn-2 tetramers in each helix. (**F**) Normalized Surface Accesible Surface Area (SASA) of R/W465 in each monomer composing the WT, HT, and FM helices. Boxes indicate 25–75 percentiles, lines within boxes indicate medians, and whiskers indicate minimum and maximum values. Violin plot indicates data distribution. *p < 0.05; **p < 0.01; ***p < 0.001 (Kruskal–Wallis and Dunn’s multiple comparisons test and one-way ANOVA for **D** and **E**, and Turkey’s multiple comparison test for **F**).

Dynamin has the propensity to oligomerize into helical arrays, and this feature has been studied using cryo-electron microscopy. To build the dyn-2 helices, we used as a template the human dyn-1 structure (PDB ID: 6DLU)^10^ along with a dyn-1 helix kindly provided by Dr. Jenny Hinshaw obtained from a cryo-electron microscopy of a liposome-associated helical polymer of dyn-1 lacking the PRD and bound to the non-hydrolysable GTP analog GMPPCP^10^. We built three different dyn-2 helix models: a dyn-2 helix with 56 WT proteins (WT helix); a helix composed of 28 WT dynamins and 28 intercalated mutant dynamins (hetero helix, HT helix), and a full mutant helix consisting of 56 mutant dynamins (FM helix). These systems of 56 dynamins with water molecules and ions contain about 8 million atoms and therefore are not yet feasible to carry full-atom MD simulations. To make this problem tractable, after vacuum minimization, we reduced the number of particles by converting full-atom helices into CG models^30^. We added GTP and Mg^2+^ to each G-domain. To simulate the neck of the vesicle during endocytosis, we built a nanotube with an outer diameter of 13.7 nm made of lipid heads. The final conformations of each helix, after 5 μs of CG simulations, are shown in Fig. 4A–C. PH domains are the closest to the lipid tube, followed by the stalk, and the G-domain. We calculated the diameter, the pitch and helix angle of each dyn-2 helix, and did not find significant differences in these parameters (Fig. 4A–D). The average helix angle during the last 100 ns of simulation in the WT helix was 5.6°, for the HT helix was 5.1°, and 6.5° for the FM helix (Fig. 4D). As the helices are composed of 14 tetramers, we measured the radius of gyration of each tetramer and found that tetramers in both HT and FM helices displayed a larger radius of gyration (Fig. 4E), suggesting conformational changes in the dynamin monomers. Since W465 exhibited a larger SASA in comparison to R465 in the full-atom dimers, we measured the SASA of R/W465 in each of the 56 monomers composing our helix models. In agreement with the full-atom results, W465 showed a larger exposition to the solvent (Fig. 4F).

### W465 perturbs interface-2 in the helix

**Figure 5:**
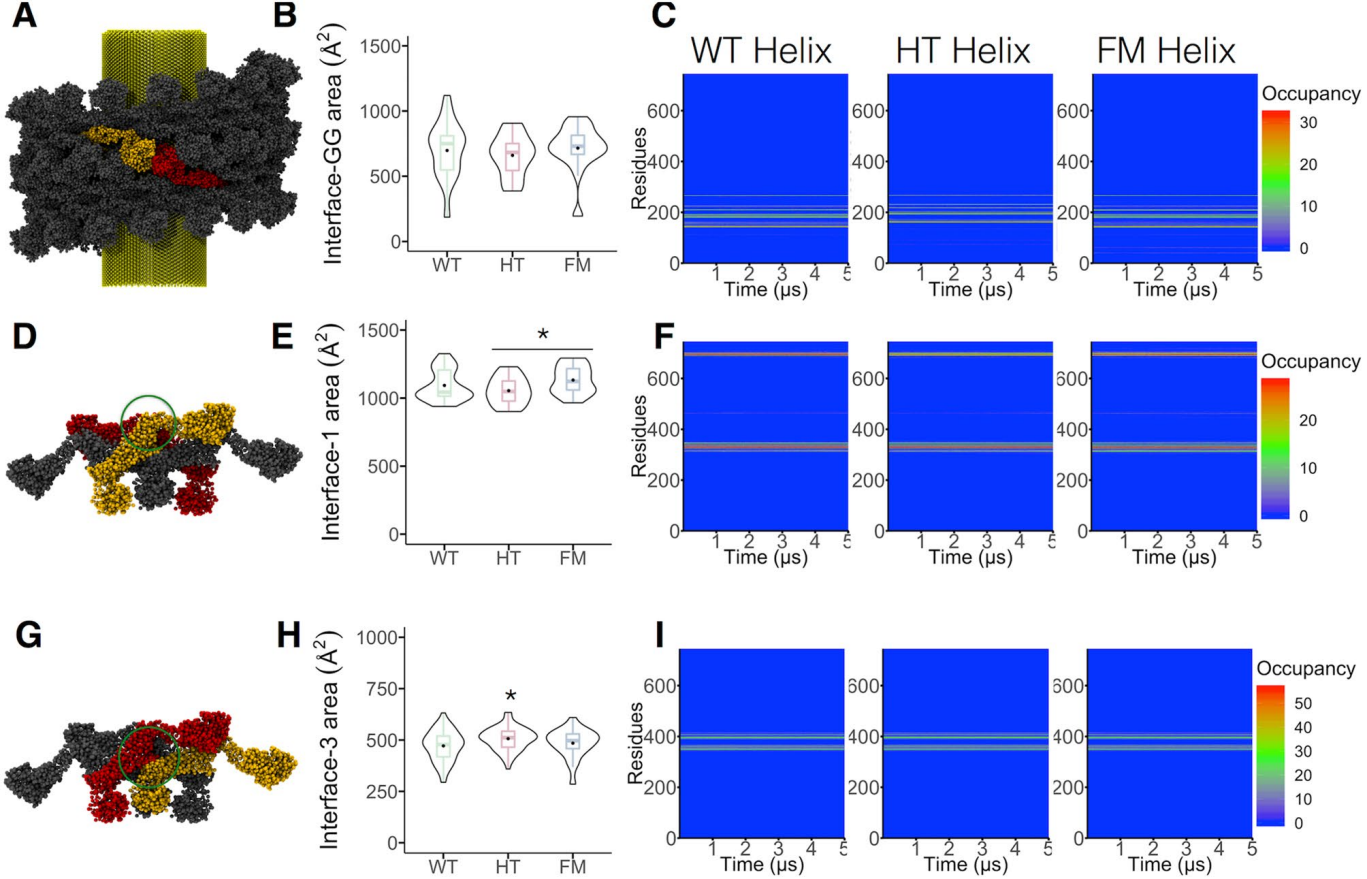
Impacts of R465W mutation on dyn-2 interfaces. (**A**) An interaction between two G domains (yellow and red) is showing in the helix. (**B**) Boxplot and violin plot of the G–G domain interface area over the last 500 ns of simulation. (**C**) Occupancy of the residues involved in the G–G domain interface during the whole trajectories. Residue occupancy was calculated as the amount of times a given residue participating in the interface is also present in the same interface by other proteins in the helix. (**D**) Representation of a tetramer (the monomers composing the interfaces are highlighted in yellow and red, whereas the other dynamins are in gray).; the circle shows the interface-1. (**E**) Plot of the interface-1 area over the last 500 ns of simulation. (**F**) Amino acids occupancy in the interface-1 during the simulation. (**G**) Scheme of interface-3 (circle) in the dyn-2 tetramer. (**H**) Interface-3 area plot over the last 500 ns of simulation. (**I**) Heatmap of the amino acids involved in interface-3 during the whole trajectories according its occupancy. Boxes indicate 25–75 percentiles, lines within boxes indicate medians, and whiskers indicate minimum and maximum values, and violin plot shows data distribution. *p < 0.05 (Kruskal–Wallis and Dunn’s multiple comparisons test for **B** and **H**, and One-way ANOVA and Turkey’s posttest for **E**).

**Figure 6:**
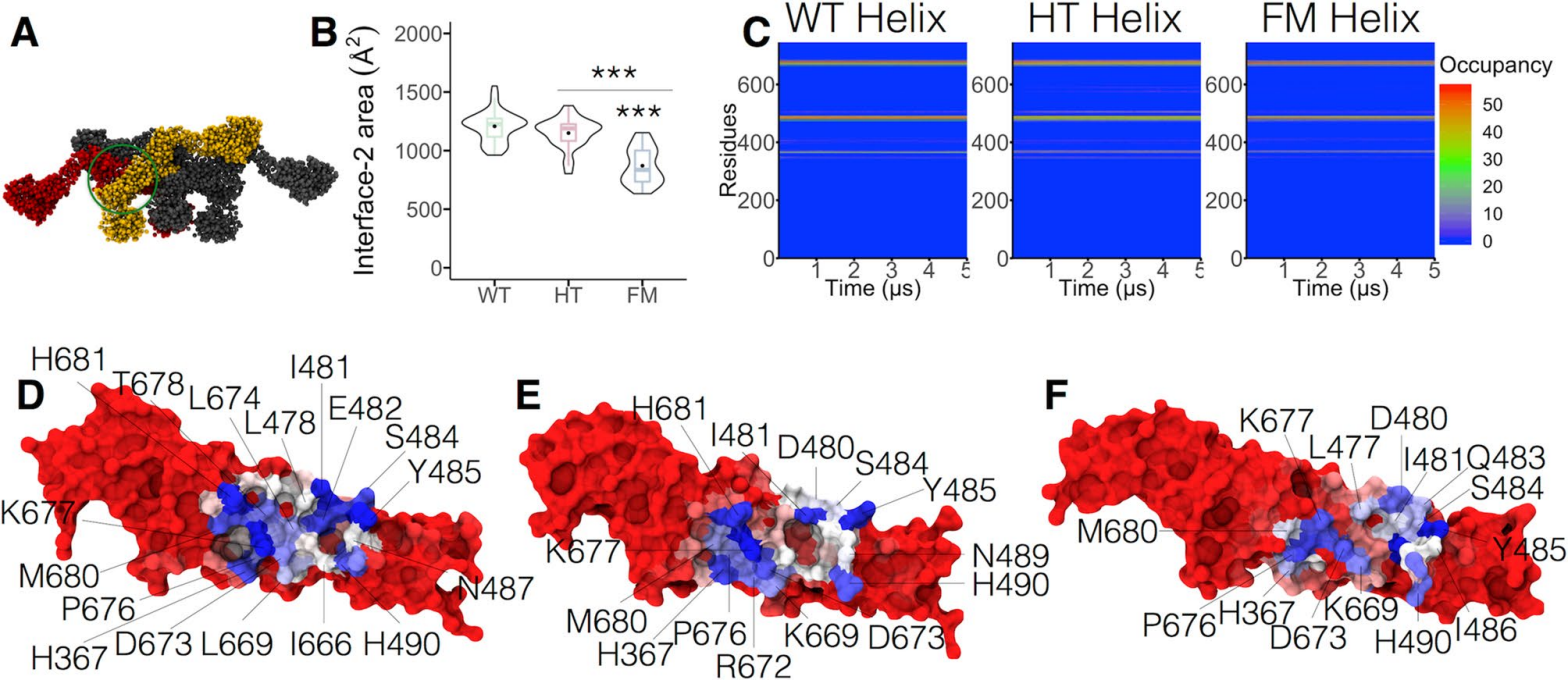
R465W modifies interface-2 in dyn-2 helices. (**A**) Representation of interface-2 (circle) in the dyn-2 tetramer. Monomers involved in this interface are highlighted in yellow and red. (**B**) Interface-2 area plot during the last 500 ns of the trajectories. (**C**) Occupancy of the amino acids involved in the interface-2 over the 5 μs of simulation. Amino acids involved in interface-2 in the (**D**) WT, (**E**) HT, and (**F**) FM helices. In blue are highlighted the residues with higher occupancy, whereas the residue with low occupancy are in red. Boxes indicate 25–75 percentiles, lines within boxes indicate medians, and whiskers indicate minimum and maximum values, and violin plot shows data distribution. ***p < 0.001 (Kruskal–Wallis and Dunn’s multiple comparisons test).

Four dynamin-dynamin interfaces were identified in the cryo-electron microscopy structure of the dyn-1 helix^8–10^: (1) the G–G domain interface that connects the dynamins of one rung with the other (Fig. 5A); (2) interface-1 located between the stalk and the BSE around residues 330 and 692^24,25^ (Fig. 5D); (3) interface-2 that takes place between two adjacent stalk in a cross-fashion^24,25^ (Fig. 6A); and (4) interface-3, which involves the loop L1N^S^ located in the stalk of one monomer with the stalk of the other dynamin^24,25^ (Fig. 5G). The fact that mutations introduced in interfaces-1 or -3 impair endocytosis highlights the functional relevance of these two interfaces^10^.

To analyze the effects of the R465W mutation on the different interfaces, we measured during the last 500 ns of CG simulation, the area included by amino acids falling within 6 Å of the adjacent monomers. Figure 5A depicts a G–G domain interaction. This interface area ranged between 186–1119 Å^2^, 387–905 Å^2^, and 194–955 Å^2^ for WT, HT, and FM helices respectively, with no significant differences between them (Fig. 5B,C). The interface-1 is represented in Fig. 5D. Its area ranged between 1093–1326 Å^2^, 901–1229 Å^2^ and 964–1294 Å^2^ in the WT, HT, and FM helices. The Kruskal–Wallis and Dunn’s multiple comparisons test revealed a significant difference only between the FM with HT helices (Fig. 5E). Analyses of residues involved in this interface showed a similar pattern between the helices, although a slightly reduced occupancy of the residues E694 and S701 was observed in the HT helix (Fig. 5F). Figure 5G shows the interface-3, which had areas that ranged between 294–632 Å^2^, 359–634 Å^2^ and 285–609 Å^2^ for the WT, HT, and FM helices, being significantly higher in HT helix in comparison to the WT helix (Fig. 5H). Analyses of the occupancy of the residues involved in this interface showed similar patterns of interaction for the three helices (Fig. 5I).

Since we observed changes in interface-2 in the dimer models (Fig. 3), we expect that this interface would be also affected in the helices. Figure 6A illustrates the interface-2. The area of this interface ranged between 961–1551 Å^2^, 803–1383 Å^2^, and 632–1153 Å^2^ for WT, HT, and FM helices, respectively, being significantly diminished in the FM helix, as compared with both WT and HT helices (Fig. 6B). Also, a different pattern of amino acid occupancy was observed for the FM helix (Fig. 6C). Among the amino acids involved in this interaction that considerably reduced their occupancy were Q474, L478, E482, N487, N489, and T678 (Fig. 6D–F). Therefore, as in the FM dimer, R465W mutation perturbs amino acid interactions that indirectly modify dynamin-2 interaction through interface-2. The reduction in the area of this interface might not impair helix stability significantly, since three other interfaces contribute to stabilizing the whole structure.

### Mutant dynamins exhibit an extended BSE in the helices

**Figure 7:**
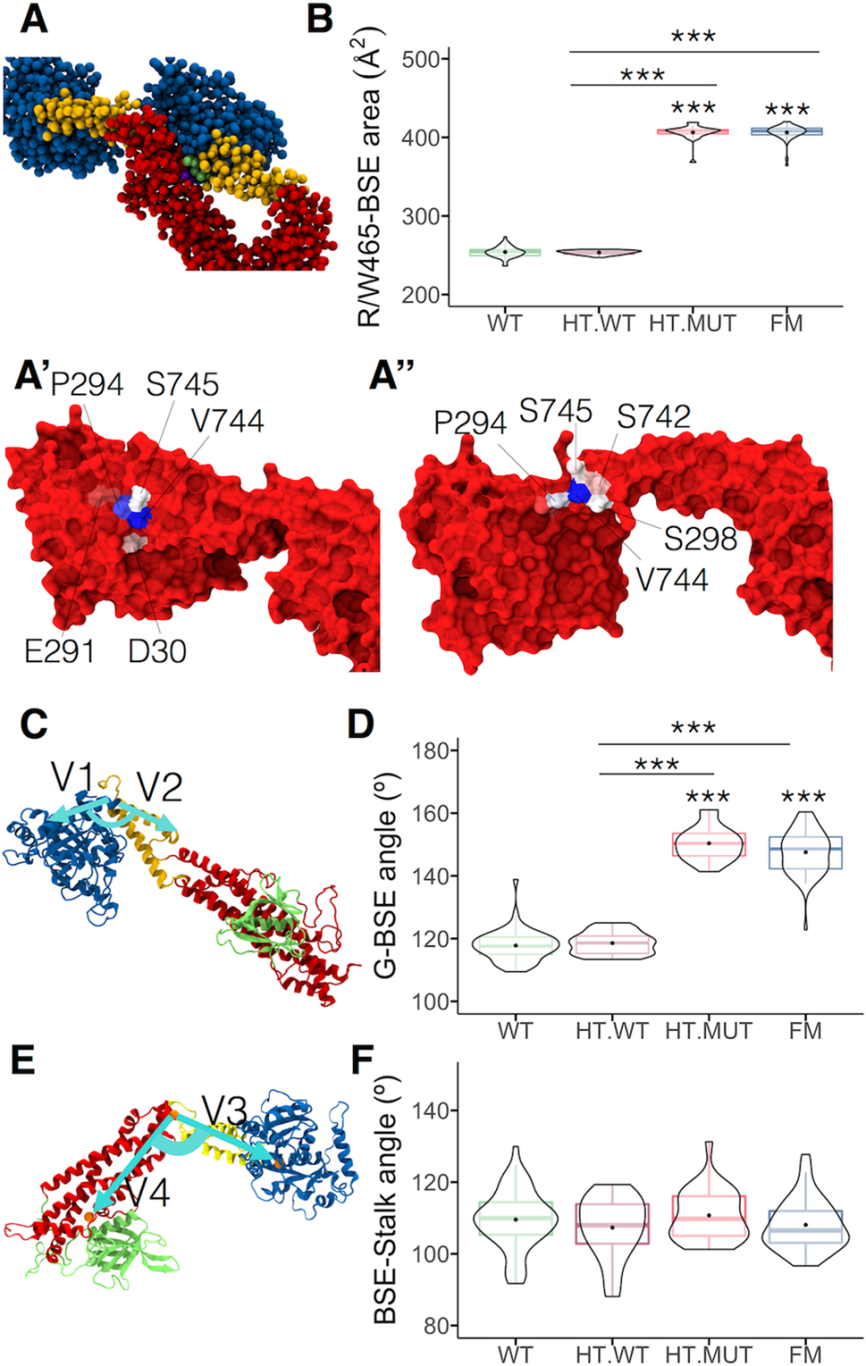
G-BSE angle is larger in mutant dynamins. **(A**) Representation of the interaction region between the residue R/W465 and the BSE of the adjacent dynamin-2. G-domain is in blue, BSE is in yellow, stalk is in red, residue 465 is shown in purple, whereas the residues of the BSE P294, S298, S742, V744, and S745 are in lime. (**A′**) BSE residues that interact with R465 in the WT monomers of the WT and HT helices. (**A″**) BSE residues that interact with W465 in the mutant monomers of the HT and FM helices. In blue are highlighted the residues with higher occupancy, whereas the residue with low occupancy are in red. (**B**) Plot of the area of interaction between the residue 465 and the BSE over the last 500 ns of simulation. (**C**) Scheme depicting the G-BSE angle. This angle is formed by the intersection of vectors V1 and V2, which correspond to the center of mass of residues 291–293. V1 ends at the center of mass of residues 277–285 segment while V2 at the center of mass of residues 299–306 segment. (**D**) Violin plot of G-BSE angles of the 56 monomers of WT and FM helices and 28 WT or mutant monomers of the HT helix (HT.WT and HT.MUT respectively). (**E**) Scheme depicting BSE-stalk angle. The BSE-stalk corresponds to the angle form by the intersection of vectors V3 and V4, which correspond to the center of mass of residues 315–321 and 702–711. Vector V3 ended at the center of the G-domain defined by segments 30–36 and 170–176. Vector V4 ended at the center of mass of residues 410–422 and 610–626. (**F**) Violin plot of BSE-stalk angles of the 56 monomers of WT and FM helices and 28 WT or mutant monomers of the HT helix (HT.WT and HT.MUT respectively). In **D** and **F** boxes indicate 25–75 percentiles, horizontal lines within boxes indicate medians, and whiskers indicate minimum and maximum angle values. ***p < 0.001 compared with WT or HT.WT (Kruskal–Wallis and Dunn’s multiple comparisons test). G domain is in blue, BSE is in yellow, stalk is in red, PH domain is in green.

Since the mutation R465W causes changes in the interactions with neighbor amino acids (Fig. 2) and, in high-order oligomeric states, R465 interacts with the BSE of an adjacent dynamin^25^, we analyzed the residues of the BSE that contact with the residue 465 and measured the interaction area (Fig. 7A). We identified that the main residues that interacted with R465 in the WT monomers of both WT and HT helices were P294, V744, and S745 (Fig. 7A′). In the mutant monomers of the HT and FM helices, in addition to these residues, W465 also contacted S298 and S742 (Fig. 7A″). The mutation also increased the area of this interaction (Fig. 7B). For WT monomers, the interaction areas ranged between 236–273 Å2 and 243–257 Å2 in the WT and HT helices, while it spanned 368–419 Å2 and 364–420 Å2 for mutant monomers in the WT and HT helices, respectively. Thus, by promoting new interactions with the BSE of the adjacent dynamin, substitutions of the arginine 465 by tryptophan may impact the helix structure at this region.

Analyses of high-resolution cryo-electron microscopy structure of human dyn-1 helices suggest that conformational changes in the BSE region are transferred across the stalk and PH domains to the membrane^10^, favoring helix constriction process^5^. Indeed, Kong et al.^10^ demonstrated that mutations in the BSE that likely disrupt such bending significantly reduce endocytosis^10^. Thus, considering the functional importance of the BSE, we analyzed the impact of the R465W mutation on its conformation, by analyzing the BSE angles with respect to the G domain and the stalk region. We defined the G domain-BSE (G-BSE) angle as the angle between vectors V1 and V2 starting at the center of mass of residues 291–293. At these amino acids the G-BSE bends. Vector V1 ended at the center of mass of residues 277–285 and V2 at the center of mass of residues 299–306 (Fig. 7C). This region corresponds to the BSE bending described by Kong et al.^10^. In Fig. 7D, the data corresponds to the average G-BSE angle of individual monomers during the last 500 ns of simulation. In the WT helix, conformations visited G-BSE angles between 109° and 138°, with an average of 117.8° (Fig. 7D). Similar angles were observed on the trajectories of WT monomers in the HT helix, averaging 118.5°. However, G-BSE angles in mutant monomers, whether in HT or FM helices, were significantly wider, averaging 150° and 147°, respectively. These results indicate that the R465W mutation favors an extended BSE conformation that has been correlated previously with impaired endocytosis^10^.

Kong et al.^10^ found that the BSE of dynamins forming the GG domain interface display asymmetry, with one BSE bent and the other unbent. We also found this asymmetry in our WT helix, with an average difference between G-BSE angles (∆) of 6.5° ± 1.3° (n = 16). This mild asymmetry was also reproduced in the FM helix (8.1° ± 1.3°; n = 16), but dramatically increased in HT helix (32° ± 1.4°; n = 16), where mutant monomers contacted WT monomers through their G domains.

The movement of the BSE with respect to the stalk is also important for membrane constriction^8^. To assess potential perturbations in the BSE-stalk angle, we defined vectors V3 and V4 starting at the center of mass of the hinge region encompassing residues 315–321 and 702–711. Vector V3 ended at the center of the G-domain defined by the center of mass of residues 30–36 and 170–176. Vector V4 ended at the center of mass of residues 410–422 and 610–626 corresponding to two alpha-helices of the stalk (Fig. 7E). Analyses of the BSE-stalk angles were like those performed for the G-BSE angles. Here, the BSE-stalk angle in the WT and both mutant helices did not differ statistically (Fig. 7F). The BSE-stalk angle of the WT helix averaged 109°, 107° for WT monomers of the HT helix, 110° for the mutant monomers of the HT helix, and 108° for the FM helix. A contributing factor may be the two flexible loops in the hinge-1 region that were not resolved in the crystallographic structure of human dyn-1 dimer^24^.

In summary, in our dyn-2 helix models the G-BSE angle was larger only in mutant dynamin monomers.

## Conclusion

Our analyses reveal that the substitution of arginine by tryptophan at position 465 of dyn-2 modifies the interactions of this residue with other amino acids in both HT and R465W dimers and helix models. In the dimer conformation, the loss of interaction and increased solvent exposure of the tryptophan in position 465 might explain the abnormal dynamin aggregation caused by this mutation^23^. These interaction changes appear to be transmitted allosterically across the protein, as the R465W mutation impacts on the dynamin-dynamin interactions by reducing the free binding energy between monomers in the HT and FM dimers (Fig. 3) and reducing the area of interface-2 in the FM helix (Fig. 6). The amino acids interaction changes induced by this mutation also favor an extended conformation of the BSE in mutant monomers in both HT and FM helices, explaining the dominance of this dynamin mutation in oligomeric structures. Extending G-BSE angle in a larger number of subunits may be related with the lethality of homozygous animal carrying this mutation^31^ in contrast to the moderated phenotype observed in heterozygous humans and mice^31,32^. Furthermore, mutations hindering BSE bending impair endocytosis^10^, suggesting that perturbations of these regions, such as we observed with R465W mutation have physiological consequences. Our findings are in agreement with the fact that skin fibroblasts from patients bearing R465W mutation and Cos-7 cells expressing this mutation exhibit disrupted dynamin fission activity^22^, a defect that is restored by allele-specific RNA interference against R465W^33^.

## Supplementary information

Supplementary Information.
