# Supplementary material for: Dynamin-2 R465W mutation induces long range perturbation in highly ordered oligomeric structures
Source: Sci Rep. 2020 Oct 23;10:18151. doi: 10.1038/s41598-020-75216-0 (PMC7584598; doi:10.1038/s41598-020-75216-0)
Supplement: Supplementary file 1 — Supplementary Information. [file 41598_2020_75216_MOESM1_ESM.pdf]

Supporting Information for

Dynamin-2 R465W mutation induces long range perturbation in highly ordered oligomeric structures

Fernando Hinostroza[1,2], Alan Neely[1], Ingrid Araya-Duran[3], Vanessa Maraboli[3], Jonathan Canan[3], Maximiliano Rojas[3], Daniel Aguayo[3], Ramón Latorre[1], Fernando González-Nilo[1,3], Ana M. Cárdenas[1].

[1]Centro Interdisciplinario de Neurociencia de Valparaíso, Universidad de Valparaíso, Gran Bretaña 1111, Valparaíso, Chile.

[2]Centro de Investigación de Estudios Avanzados del Maule, Universidad Católica del Maule, Av. San Miguel 3605, Talca, Chile.

[3]Center for Bioinformatics and Integrative Biology, Universidad Nacional Andrés Bello, Av. República 330, Santiago, Chile.

*Correspondence:

Index

Supplemental Figure 3

Methods 5

- **Full-atom molecular dynamics simulations** 5
- **Free binding energy calculation** 5
- **Coarse-grained Molecular Dynamics simulations** 5
- **Solvent Accesible Surface Area Calculation** 6
- **Statistics** 6

References 7

Supplemental Figure

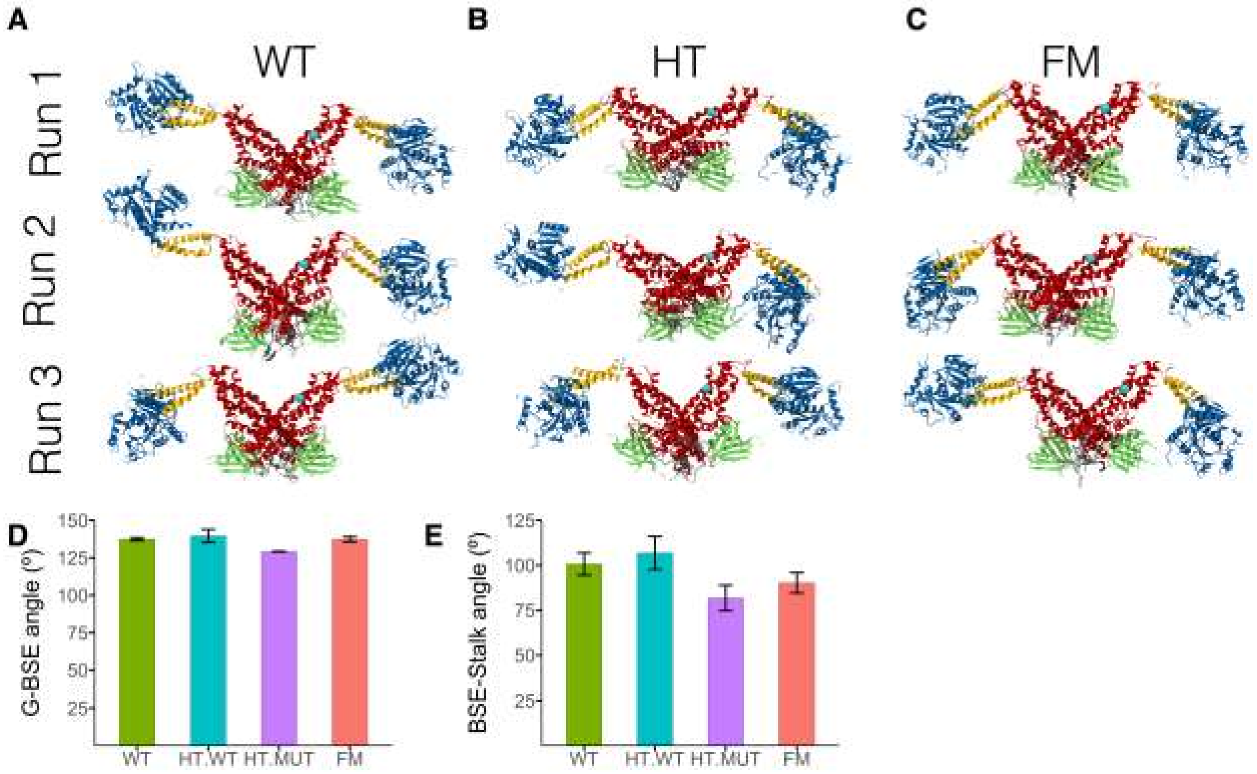

Figure S1: *Dynamin-2 dimer models*. Three replicates were run for each dimer system. G domains are in blue, bundle signaling elements (BSEs) in yellow, stalks in red, and PH domains in green. Final dynamin-2 dimer conformations of each replicate after 200 ns of simulation of the WT (**A**), HT (**B**), and FM (**C**) systems. The residue 465 is displayed as cyan dot. Measurement of the G-BSE (**D**) and BSE-stalk (**E**) angles showed that there are no significant structural differences between the three conditions (One-way ANOVA). The vector used to calculate these angles were the same that those we used in the Coarse-grained analysis to measure the G-BSE and BSE-stalk angles (see Results and Discussion section).

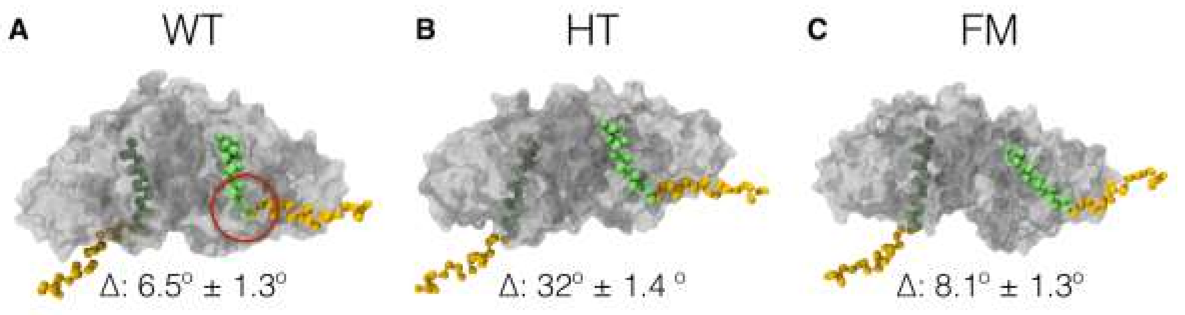

Figure S2: *G-BSE angle asymmetries in the Dynamin-2 helices*. The G-G domain interactions are shown for WT (**A**), HT (**B**), and FM (**C**) helices. The asymmetry of the G-BSE angle was calculated as the difference (Δ) between the averaged G-BSE angles of each dynamin-2 composing the G-G domain dimer. G domain is shown in transparent white and green, and BSE in yellow. G-BSE angle is circle in red.

Methods

Construction of dynamin-2 homology model

To create the dynamin-2 (dyn-2) homology model, human dynamin-1 (dyn-1) crystallographic structure (PDB ID: 3SNH)[1] was used as a template (Suppl. Fig. 1A). Amino acids 1-5, 63-64, 110-112, 143-149, 347-356, 394-404, 446-447, 500-517, 534-537, 578-581, 632-652, and 710-716 are not resolved in the human dyn-1. We completed those regions using Modeller v9.10 software[2] and selected the model with the lowest DOPE potential. Next, the created loops were relaxed through 1 ns MD simulation (Suppl. Fig. 1B) and built the dyn-2 homology model using the relaxed human dyn-1 as a template by the internal coordinate mechanics (ICM) method with Modeller v9.10[3] software. We chose the best model according to the DOPE potential that was relaxed using CHARMM36 force field[4] and NAMD v2.0 software[5] (Suppl. Fig. 1C).

Full-atom molecular dynamics simulations

For full-atom MD, we mutated the WT protein by using the Mutator plugging of Visual Molecular Dynamics[6]. The arginine 465 was exchanged for a tryptophan. Next, we built the WT HT and R465W dimer systems. Each system was added to a water box. The hydrated systems were neutralized with NaCl at a concentration of 150 mM. The full systems were relaxed through molecular dynamics (MD) simulations using the AMBER suit[7]. MD was performed with the ff14SB force field[8]. The temperature was set at 310 K using the Langevin thermostat with the isobaric-isothermal (NPT) ensemble. We fixed the pressure at 1 atm. The integration of the equation of motion of Newton was performed with the verlet algorithm using a 2 ps timestep. For non-bonded interactions, a cutoff of 10 nm was used. Each system was subjected to energy minimization using periodic boundary conditions. Three replicas of each system were run for 200 ns.

Free binding energy calculation

Free Binding Energy calculations were obtained with the MM/GBSA by using AmberTools18[7].

Coarse-grained Molecular Dynamics simulations

We used the human dyn-1 coordinates (PDB ID: 6DLU)[9] along with a dyn-1 helix kindly provided by Dr. Jenny Hinshaw as a template to build the dyn-2 helices. We built: a dyn-2 helix with 56 WT proteins (WT helix); a helix composed of 28 WT dynamins and 28 mutated intercalated dynamins (HT helix), and a mutated helix consisting of 56 mutant dynamins (R465W helix). To reduce the number of particles, after vacuum minimization, we converted full-atom helices into coarse-grained (CG) models[10] using Martini force field v2.2. These CG models were reduced to ~500.000 particles. We applied elastic networks to ensure the CG dyn-2 proteins behave properly. We added a GTP and a magnesium ion on each dynamin-2 G-domain. Each GTP and Mg+2 were under distance restraints to avoid the separation from the G-domain. GTP molecules were composed of SC1 (TN0), SC2 (TG2), SC3 (TG3), SC4 (TNa), BB2 (SN0), BB3 (SC2), and three BB1 (Q0) coarse-grained beads (Uusitalo et al., 2015), and a MG+ (Qd) beads for Mg^{+2}. We also added a nanotube with an

outer diameter of 13.7 nm composed of lipid heads to reduce computational cost and simulate the neck of the vesicle during endocytosis. The glycerol molecules from the lipid nanotube were under position restraints of 1000 kJ/mol/nm^2 during the simulations. We minimized each system in vacuum for 50.000 steps. Next, each system was solvated with non-polarizable CG water molecules BP4[11] and ionized them with 150 mM of NaCl. To prevent freezing of water molecules, we added anti-freeze BP4 beads[11]. Then, each system was submitted to 5 µs of MD simulation in an NPT ensemble. We fixed the temperature at 310 K using the v-rescale thermostat and the pressure was fixed at 1 atm using the Parrinello-Rahman barostat[12]. Electrostatics interactions were calculated using the Reaction-field method. Van der Waals interactions were calculated using the Verlet cut-off-scheme. The Newton's equation of motion was integrated with the leapfrog algorithm with a timestep of 10 fs using Gromacs software v.5.0[13] for all the CG simulations carried out with periodic boundary conditions.

Solvent Accesible Surface Area Calculation

To calculate the SASA, the radius of the amino acid, that is Arg465 or Trp465, were used to find the points of the residue exposed to the solvent. Only the points near the residue Arg465 or Trp465 were considered. To prevent that internal pockets or protein voids affects SASA calculation, we used the restrict option. The SASA calculations use as a reference the SASA of a specific residue between 2 alanine by each side, which means to use the sequence AAWAA, which represents the 100% of the exposition of a residue in water.

Statistics

For comparing the three different conditions we used ANOVA analysis and Turkey post-test for data with a normal distribution, and Kruskal-Wallis and Dunn's post-test for non-normal distribution datasets. We plotted the data with R Studio software.

References

1. Faelber, K.; Posor, Y.; Gao, S.; Held, M.; Roske, Y.; Schulze, D.; Haucke, V.; Noe, F.; Daumke, O., Crystal structure of nucleotide-free dynamin. *Nature* **2011,** *477* (7366), 556-60.
2. Sali, A.; Blundell, T. L., Comparative protein modelling by satisfaction of spatial restraints. *J Mol Biol* **1993,** *234* (3), 779-815.
3. Cardozo, T.; Totrov, M.; Abagyan, R., Homology modeling by the ICM method. *Proteins* **1995,** *23* (3), 403-14.
4. Huang, J.; MacKerell, A. D., Jr., CHARMM36 all-atom additive protein force field: validation based on comparison to NMR data. *J Comput Chem* **2013,** *34* (25), 2135-45.
5. Phillips, J. C.; Braun, R.; Wang, W.; Gumbart, J.; Tajkhorshid, E.; Villa, E.; Chipot, C.; Skeel, R. D.; Kale, L.; Schulten, K., Scalable molecular dynamics with NAMD. *J Comput Chem* **2005,** *26* (16), 1781-802.
6. Humphrey, W.; Dalke, A.; Schulten, K., VMD: visual molecular dynamics. *J Mol Graph* **1996,** *14* (1), 33-8, 27-8.
7. D.A. Case, I. Y. B.-S., S.R. Brozell, D.S. Cerutti, T.E. Cheatham, III, V.W.D. Cruzeiro, T.A. Darden, R.E. Duke, D. Ghoreishi, M.K. Gilson, H. Gohlke, A.W. Goetz, D. Greene, R Harris, N. Homeyer, S. Izadi, A. Kovalenko, T. Kurtzman, T.S. Lee, S. LeGrand, P. Li, C. Lin, J. Liu, T. Luchko, R. Luo, D.J. Mermelstein, K.M. Merz, Y. Miao, G. Monard, C. Nguyen, H. Nguyen, I. Omelyan, A. Onufriev, F. Pan, R. Qi, D.R. Roe, A. Roitberg, C. Sagui, S. Schott-Verdugo, J. Shen, C.L. Simmerling, J. Smith, R. Salomon-Ferrer, J. Swails, R.C. Walker, J. Wang, H. Wei, R.M. Wolf, X. Wu, L. Xiao, D.M. York and P.A. Kollman, AMBER 2018. **2018**, Universidad de California, San Francisco.
8. Maier, J. A.; Martinez, C.; Kasavajhala, K.; Wickstrom, L.; Hauser, K. E.; Simmerling, C., ff14SB: Improving the Accuracy of Protein Side Chain and Backbone Parameters from ff99SB. *J Chem Theory Comput* **2015,** *11* (8), 3696-713.
9. Chappie, J. S.; Mears, J. A.; Fang, S.; Leonard, M.; Schmid, S. L.; Milligan, R. A.; Hinshaw, J. E.; Dyda, F., A pseudoatomic model of the dynamin polymer identifies a hydrolysis-dependent powerstroke. *Cell* **2011,** *147* (1), 209-22.
10. Monticelli, L.; Kandasamy, S. K.; Periole, X.; Larson, R. G.; Tieleman, D. P.; Marrink, S. J., The MARTINI Coarse-Grained Force Field: Extension to Proteins. *J Chem Theory Comput* **2008,** *4* (5), 819-34.
11. Marrink, S. J.; Risselada, H. J.; Yefimov, S.; Tieleman, D. P.; de Vries, A. H., The MARTINI force field: coarse grained model for biomolecular simulations. *J Phys Chem B* **2007,** *111* (27), 7812-24.
12. M. Parrinello, A. R., Polymorphic transitions in single crystals: a new molecular dynamics method. *J. Appl. Phys.* **1981,** *52*, 7182-7190.
13. Berendsen, H. J. C. V., D.; Vandrunen, R., Gromacs - a Message-Passing Parallel Molecular-Dynamics Implementation. *Comput Phys Commun* **1995,** *22* (21), 2695-6.
